# Interaction of a Novel Alternatively Spliced Variant of *HSD11B1L* with Parkin Enhances the Carcinogenesis Potential of Glioblastoma: Peiminine Interferes with This Interaction

**DOI:** 10.3390/cells12060894

**Published:** 2023-03-14

**Authors:** Ru-Huei Fu, Syuan-Yu Hong, Chia-Wen Tsai, Shih-Ping Liu, Shao-Chih Chiu, Meng-Zhen Wu, Woei-Cherng Shyu, Shinn-Zong Lin

**Affiliations:** 1 Graduate Institute of Biomedical Sciences, China Medical University, Taichung 40402, Taiwan; 2 Translational Medicine Research Center, China Medical University Hospital, Taichung 40447, Taiwan; 3 Department of Medicine, School of Medicine, China Medical University, Taichung 40447, Taiwan; 4 Division of Pediatric Neurology, China Medical University Children’s Hospital, Taichung 40447, Taiwan; 5 Department of Nutrition, China Medical University, Taichung 40402, Taiwan; 6 Buddhist Tzu Chi Bioinnovation Center, Tzu Chi Foundation, Hualien 97002, Taiwan; 7 Department of Neurosurgery, Buddhist Tzu Chi General Hospital, Hualien 97002, Taiwan

**Keywords:** glioblastoma, alternative splicing, hydroxysteroid 11-β-dehydrogenase 1 like gene (HSD11B1L), proliferation, migration, invasion, parkin, peiminine

## Abstract

Glioblastoma (GBM) is a primary brain tumor of unknown etiology. It is extremely aggressive, incurable and has a short average survival time for patients. Therefore, understanding the precise molecular mechanisms of this diseases is essential to establish effective treatments. In this study, we cloned and sequenced a splice variant of the hydroxysteroid 11-β dehydrogenase 1 like gene (*HSD11B1L*) and named it HSD11B1L-181. HSD11 B1L-181 was specifically expressed only in GBM cells. Overexpression of this variant can significantly promote the proliferation, migration and invasion of GBM cells. Knockdown of HSD11B1L-181 expression inhibited the oncogenic potential of GBM cells. Furthermore, we identified the direct interaction of parkin with HSD11B1L-181 by screening the GBM cDNA expression library via yeast two-hybrid. Parkin is an RBR E3 ubiquitin ligase whose mutations are associated with tumorigenesis. Small interfering RNA treatment of parkin enhanced the proliferative, migratory and invasive abilities of GBM. Finally, we found that the alkaloid peiminine from the bulbs of *Fritillaria thunbergii* Miq blocks the interaction between HSD11B1L-181 and parkin, thereby lessening carcinogenesis of GBM. We further confirmed the potential of peiminine to prevent GBM in cellular, ectopic and orthotopic xenograft mouse models. Taken together, these findings not only provide insight into GBM, but also present an opportunity for future GBM treatment.

## 1. Introduction

Glioblastoma multiforme (GBM) is the most common and malignant primary brain tumor in adults. Almost 100% of patients relapse and the median survival time is less than tens of months. Due to its high invasiveness and heterogeneity, GBM is still extremely difficult to relieve with surgery, targeted/immunotherapy or radiation/chemotherapy [1,2]. At present, analysis of genetic variation shows that the formation of GBM is closely related to a disorder of intracellular signal transduction and uncontrolled cell cycles. Therefore, to accurately clarify the exact molecular mechanism of cancer development, then to establish effective prevention and treatment strategies, is the direction of current efforts in this field [3].

Alternative splicing (AS) is a mechanism by which eukaryotic cells express many heterogeneous products with a limited number of genes. It is a post-transcriptional modification of mRNA. AS causes exons or a small part of introns of pre-mRNA to be selectively joined to form mature mRNA [4]. Sometimes the scope of exon sequence definition also changes (alternative 5′ splice sites and/or alternative 3′ splice sites). The result is a transcript with different RNA regulatory elements (stability and translation efficiency) or protein coding sequences [5]. AS mainly uses or restricts specific splicing sites through the cooperative combination of different trans-splicing factors and various cis-regulatory elements in the transcript [6]. AS is involved in different developmental stages, tissue types and stress responses in mammals. Ninety percent of human genes are regulated by AS, and its abnormality can cause many diseases, including cancer [7] and neurodegenerative diseases [8].

Part of the GBM may be related to abnormal AS mechanism. Iyoda et al. previously discovered that a splicing variant of the Tenascin (TN)-C molecule contains the TNIIIA2 fragment in the fibronectin III-A2 type domain, which can promote survival/growth, metastasis, and invasion of GBM cells by regulating the activity and persistence of β1 integrin related to cell adhesion [9]. In three estrogen-related receptor β (ERR-β) protein products, ERR-β short form (ERR-βsf), ERR-β2 and ERR-β, exon 10 deletion is caused by AS at the 3′end of pre-mRNA. Studies have shown that the shorter ERR-βsf subtype plays a role in aging and G1 cell cycle arrest, contrary to the function of the ERR-β2 subtype in G2/M cell cycle arrest and inducing apoptosis. ERR-β2 interacts with the actin nucleation-promoting factor cortactin and blocks GBM cell migration. The enhanced expression of ERR-β2 can be induced by the ERR-β agonist, and the inhibition of splicing-regulatory cdc2-like kinase leads to the suppression of the growth and migration of GBM cells and intracranial tumors [10]. Li et al. found that arginine/serine-rich protein 1 (RSRP1) mediates the spliceosome assembly, leading to skipping of exon 18 of PARP6 to form truncated oncogenic PARP6-s. This isoform cannot effectively inhibit the activity of NF-κB, and finally promotes GBM cell invasion and anti-apoptotic mesenchymal phenotype [11]. 

A comprehensive and systematic analysis of AS events and related *trans*-acting splicing factors has been applied to GBM patients for prognostic and therapeutic purposes [12,13,14,15,16,17]. Li and Guo analyzed the RNA-seq and AS events data (TCGA SpliceSeq data) of 132 GBM samples downloaded from The Cancer Genome Atlas (TCGA). From 45,610 AS events, 416 AS events related to GBM survival rate were identified, and an AS association network of 54 AS changes and 94 factors associated with splicing was established [18]. Among them, the AS event of the hydroxysteroid 11-beta dehydrogenase 1 like gene (*HSD11B1L*) has aroused our interest. HSD11B1L is known to be mainly expressed in the brain of higher mammals and its function may be related to the regulation of the activity of the stress hormone glucocorticoid (GC) [19]. GC has been revealed to increase chemoresistance, suppress microglial immune responses against cancer and enhance glioma growth in vivo [20]. We further used molecular cloning techniques to confirm the existence of an AS variant in GBM8401 cells and named it HSD11B1L-181. Compared with HSD11B1L, HSD11B1L-181 deleted the N-terminal 134 amino acid fragment and replaced the C-terminal 63 amino acid fragment with a different 92 amino acid fragment. HSD11B1L-181 displays significant carcinogenesis potential in GBM. The results show that HSD11B1L-181 is specifically higher expressed in various GBM cell lines. We also showed that HSD11B1L-181 overexpression significantly augmented the proliferation, migration and invasion abilities of GBM 8401 cells. The downregulation of the expression of HSD11B1L-181 significantly inhibited the progression of GBM 8401. Furthermore, in order to explore the possible mechanism of the effect of HSD11B1L-181 on GBM, we screened the human GBM cDNA expression library by yeast two-hybrid, and confirmed that E3 ubiquitin ligase parkin is a specificity target of HSD11B1L-181 by additional co-immunoprecipitation and immunofluorescence staining. Studies have shown that the deletion of parkin is closely related to the occurrence of cancer [21]. We abolished the expression of parkin in GBM8401 cells and significantly augmented the oncogenic potential of those cells. Therefore, we believe that HSD11B1L-181 may promote the development of GBM by combining and interfering with the activity of parkin. Finally, we used a yeast and a cellular model to assess the ability of several phytocompounds already in our laboratory to interfere with the interaction between HSD11B1L-181 and parkin. We found that the alkaloid peiminine (PMN) from the bulbs of *Fritillaria thunbergii* Miq can obstruct the interaction between HSD11B1L-181 and parkin. It has also been shown to prevent GBM growth in both subcutaneous xenograft and orthotopic xenograft mouse models.

## 2. Materials and Methods

### 2.1. GBM and Other Cancer Cell Lines, Primary Cells, Culture Medium and Reagents

GBM and other cancer cell lines were obtained from the Biological Resource Collection and Research Center (BCRC, Hsinchu, Taiwan). Primary cells of the human brain were acquired from ScienCell Research Laboratories (San Diego, CA, USA). Media and related reagents were acquired from Gibco, ThermoFisher Scientific (Waltham, MA, USA). The GBM8401 was routinely maintained in RPMI 1640 with 10% fetal bovine serum and 1% antibiotics at 37 °C and 5% CO_2_. All chemicals were obtained from Sigma-Aldrich (St. Louis, MI, USA) unless otherwise noted.

### 2.2. RT-PCR

To identify the expression of HSD11B1L-WT and HSD11B1L-181 transcripts, we first isolated the total RNA of GBM8401 by TRIzol reagent (Invitrogen, Carlsbad, CA, USA) according to the manufacturer’s protocol. Next, we implemented reverse transcription using the SuperScript III One-Step RT-PCR system (Invitrogen). Briefly, the mRNA and regents were incubated at 55°C for 60 min. Then the PCR reaction was conducted: the first cycle is 2 min at 94 °C; the following steps are performed for 35 cycles at 94 °C for 15 s, 60 °C for 30 s and 68 °C for 30 s; the last cycle is at 68 °C for 5 min. The primer pair sequence design was 5′-TGTGAGCTACGTGCAACTGA -3′ (forward) and 5′-TGCAAGTCCCCGACAAAGG -3′ (reverse). Finally, the products of amplified DNA and markers can be visualized by separation by agarose gel electrophoresis and staining with ethidium bromide. The internal loading control is β-actin. The primer pair sequence was 5′- CACAGAGCCTCGCCTTTGC -3′ (forward) and 5′- AGGAATCCTTCTGACCCATGC -3′ (reverse). All primers were purchased from Tri-i Biotech (Taipei, Taiwan).

### 2.3. Western Blot

RIPA lysis and extraction buffer (ThermoFisher Scientific) containing halt protease and phosphatase inhibitor cocktail was used for the preparation of cell lysates. Fifty micrograms of sample (lysate) were resolved by 10–12.5% SDS-PAGE (Amresco, Solon, OH, USA) and then were transferred to PVDF membranes (Millipore Corp., Burlington, MA). Membranes were washed and reacted overnight at 4 °C with HSD11B1L (ThermoFisher Scientific), c-Myc or parkin (Cell Signaling Technology, Beverly, MA, USA) antibodies. Finally, membranes were washed and HRP-conjugated secondary antibodies were added (Enzo Life Sciences, Farmingdale, NY, USA). After 1 h incubation at room temperature, blots were detected by the UVP BioSpectrum Imaging Device (Upland, CA, USA) via Amersham Enhanced Chemiluminescence Regent (Piscataway, NJ, USA).

### 2.4. Construction and Transfection of Plasmid

The coding DNA sequences of HSD11B1L-WT and HSD11B1L-181 were synthesized by Genomics (Taipei, Taiwan) and ligated with the pcDNA 3.1/myc-His vector (Invitrogen). The reagent of Lipofectamine 2000 (Invitrogen) was employed for transfection, and the expression plasmid was transiently transfected into GBM8401 cells according to the manufacturer’s instructions, and then the successfully transfected population was selected via geneticin (G418) (Invitrogen) for subsequent experiment.

### 2.5. Cell Proliferation Assay

CellTiter Blue Cell Viability Assay kit (Promega, Madison, WI, USA) was used to assess cell proliferation ability. Briefly, evaluated cells were cultured in 96-well plates (4 × 10^3^/well). Then, CellTiter-Blue^®^ Reagent was added to the well at 12, 24, 36, 48, 60 and 72h, followed by incubation at 37 °C for 2 h. Finally, viable cells were quantified by fluorescence intensity (λex = 560; λem = 590 nm) via a SpectraMax M2 microplate reader (Molecular Devices, Silicon Valley, CA, USA).

### 2.6. Scratch Test for Cell Migration

The migratory ability of GBM cells was determined by a scratch test. Briefly, cells were cultured in 6-well plates overnight, and then scraped with a micropipette tip to create gaps (wounds). Twelve hours later, employing an Axio Observer inverted fluorescence microscope (Carl Zeiss MicroImaging GmbH, Göttingen Germany) to image the scratched cell monolayers at 100× magnification, the average width of the gap that cells had migrated into was calculated via AxioVision software 4.8 (Carl Zeiss, Göttingen, Germany). The average width of each gap was calculated by the top, middle and bottom positions in the microscopic field.

### 2.7. Cell Invasion Assay

GBM cell invasion was measured and quantified in vitro using the InnoCyte Cell Invasion Assay Kit (Merck Ltd., Taipei, Taiwan) according to the manufacturer’s instructions. Briefly, 1.5 × 10^5^ cells in serum-free medium (300 µL) were cultured in the upper chamber (insert) and into the lower chamber was added 10% fetal bovine-serum-containing medium (500 µL). After 24 h of incubation, the medium of upper chamber was removed and the inserts placed into new wells with fluorescent Calcein-AM staining solution (500 µL, diluted 1:300 with cell detachment buffer). After 1 h, 200 µL of detached cells were transferred to a black 96-well plate and fluorescence (λex = 485; λem = 520 nm) was measured using a SpectraMax M2 microplate reader (Molecular Devices). Additionally, cells passing through the basement membrane matrix on the inserts (invasive cells) were visualized using 0.1% crystal violet staining.

### 2.8. Transient Transfection of Small Interfering RNA of Parkin

Transient transfection of targeting siRNA (75 nM) or non-targeting siRNA (control) was implemented by Lipofectamine 2000 reagent (Invitrogen) following the manufacturer’s instructions. Briefly, GBM8401 cells were cultured in 6-well plate (2.0 × 10^5^ cells). When 70% confluency was reached, we proceeded to transfection for 24 h. The sequences of siRNA for HSD11B1L-181 were (1) 5′-CAUAGCCGGGGUGGUCACU-3′. For human parkin was (1) 5′-AAGGACCGCAAAGUGUUUGUG-3′. Non-silencing RNA duplex 5′-AAUUCUCCGAACGUGUCACGU-3′ was used as a control as designated by the manufacturer.

### 2.9. Interaction Screening by Yeast Two-Hybrid

We used the Matchmaker GAL4-based two-hybrid system (Clontech, Mountain View, CA, USA) to screen the candidate partners involved in HSD11B1L-181 interaction. First, the “bait” of HSD11B1L-181 was inserted into the pGBKT7 vector and pre-transformed into the AH109 strain of *S. cerevisiae*. The human cDNA expression library from GBM8401 (Clontech) as a prey (pGADT7 vector) was constructed and expressed in the Y187 strain. Y187 strains carrying the pre-transformed library were mated with bait AH109 strains to generate diploids. Interactions of HSD11B1L-181 with candidate proteins were determined based on the growth of diploids on SD/-Ade/-His/-Leu/-Trp/X-α-gal plates. To identify positively interacting genes, positive diploids were cultured on a liquid medium of SD/-Ade/-His/-Leu and then plasmids were isolated by Zymoprep™ Yeast Plasmid Miniprep I (Zymo Research Corporation, Irvine, CA, USA). Finally, the prey cDNAs were sequenced and analyzed.

### 2.10. Yeast Two-Hybrid Assays

The full-length and partial coding DNA sequences of HSD11B1L-181 and parkin were inserted into pGBKT7 and pGADT7 vectors, respectively, and then were transformed into AH109 and Y187 strains, respectively. According to Section 2.9, yeast two-hybrid assays were implemented. Additionally, reverse yeast two-hybrid assay further confirmed the specific interaction between HSD11B1L-181 and parkin protein in vivo.

### 2.11. Co-Immunoprecipitation for HSD11B1L-181 and Parkin

Coding DNA fragments of HSD11B1L-181 and parkin were inserted into pCMV-HA and pCMV-Myc (Clontech, Mountain View, CA, USA), respectively. Next, using Lipofectamine 2000 reagent (Invitrogen), both plasmids were delivered into 293T cells. After 48 h, those cells were incubated in EBC lysis buffer. Then the lysate was centrifuged to collect the soluble supernatant. Subsequently, using protein G-sepharose, the supernatant was cleared and anti-HA antibody (rabbit or mouse) or normal immunoglobulin G (rabbit or mouse) were employed to perform the immunoprecipitation at 4 °C for 2 h, followed by incubating the protein G-sepharose beads for another 1 h. Finally, the immunoprecipitated complex was washed and detected by Western blotting by the anti-cMyc antibody (mouse or rabbit). All antibodies in this analysis were purchased from Cell Signaling Technology (Beverly, MA, USA). For reverse immunoprecipitation, the coding DNA fragments of HSD11B1L-181 and parkin were ligated with pCMV-Myc and pCMV-HA, respectively. Immunoprecipitation analysis was then performed as described previously.

### 2.12. Immunofluorescence Staining Analysis

GBM cells expressing HSD11B1L-181 were grown on poly-L-lysine-coated coverslips. When reaching 80% confluence, cells were washed and fixed with 4% paraformaldehyde. After washing, mouse anti-myc and rabbit anti-parkin antibodies (Cell Signaling Technology) were added and reacted at 4 °C. On the second day, samples were washed, and then goat anti-mouse (green fluorescence labeled) and goat anti-rabbit (red fluorescence labeled) secondary antibody (Invitrogen) were added at 25 °C. After 1 h, samples were washed and stained with DAPI. The signal was observed by a Zeiss Axio Imager A1 fluorescence microscope (Carl Zeiss MicroImaging GmbH, Göttingen, Germany). 

### 2.13. PMN Treatment, Yeast Two-Hybrid Based Growth Assay

Synthetic PMN (mol. wt. 429.64, 98% purity) was acquired from Rainbow Biotechnology Co. Ltd. (Shilin, Taipei, Taiwan) and dimethyl sulfoxide (DMSO) was used as a solvent (1 M). The amount of DMSO used in all experiments was 0.1%. The diploid yeasts, including BD-/AD-, BD-p53/AD-T, BD-HSD11B1L-181-H2/AD-parkin-P2 and BD-parkin-P2/AD-HSD11B1L-181-H2, were grown until they reached log or mid-log phase in SD/-Leu/-Trp broth at 30°C. In the spot assay, the optical density (OD_600_) of all cultures was normalized, serially diluted and spotted using a pipette (10 μL) into 0, 1, 5 or 10 μM PMN containing SD/-Leu/-Trp plates or SD/-Ade/-His/-Leu/-Trp plates, and then grown at 30 °C for 3 days. In absorbance measurements, the diploid yeast cultures of each group were normalized and incubated on liquid medium of 0, 1, 5 or 10 μM PMN-containing SD/-Leu/-Trp or SD/-Ade/-His/-Leu/-Trp/X-α-gal. OD values were measured every 12 h for 48 h.

### 2.14. TUNEL Assay

In situ apoptosis of PMN-treated (24 h) GBM cells was detected with a Click-iT™ Plus TUNEL Assay kit (invitrogen). Cells grown in coverslips were fixed and permeabilized. Subsequently, samples were incubated in Tdt reaction buffer for 10 min and Tdt reaction mixture for 60 min at 37 °C. The following samples were determined by the Click-iT™ plus TUNEL reaction cocktail for 30 min (dark) and a Zeiss Axio Imager A1 fluorescence microscope (Carl Zeiss MicroImaging GmbH) was employed to image.

### 2.15. Subcutaneous Tumor Xenograft Model of Mouse

All mice studies were performed consistent with the Guidelines for the Care and Use of Institutional Animals of China Medical University and the Guidelines for the Care and Use of Laboratory Animals of the National Institutes of Health. Mice husbandry, care and application protocols for investigational processes were approved by the Institutional Animal Care and Use Committee of China Medical University (permit number: CMUIACUC-2022-248). Male nude mice of BALB/c (2 months old, 20 to 25 g) were gained from the National Laboratory Animal Center (Taipei, Taiwan). Fifty μL GBM8401 cells (2 × 10^6^) were mixed with Matrigel™ Basement Membrane Matrix (50 μL, BD Biosciences, San Jose, CA, USA) and inoculated subcutaneously on the right side of mice. We assessed tumor growth using Vernier calipers. Tumor volume (V) was measured as V (mm^3^) = (D1^2^ × D2)/2. D1 and D2 were defined as the shortest and longest tumor diameters, respectively. Different treatments were performed after tumors had grown to approximately 25 ± 1 mm^3^ (day 0). All tumor-bearing mice were randomly divided into five groups (*n* = 6): control group (saline), DMSO and PMN (0.25, 0.5, and 1.0 mg/kg). Mice were treated accordingly by intraperitoneal injection (50 μL) on days 4, 8, 12 and 16. During treatment, body weight and tumor size were measured every day. After 1 month, the animals were sacrificed by euthanasia, and the tumors were separated and weighed. Moreover, in order to record the survival time in this study, mice (*n* = 5) were included in each group as in the previous experiment. Survival time to day 90 was assessed. Finally, we used Kaplan—Meier statistics and log-rank test to calculate the median value of survival of mice in all treatment groups.

### 2.16. Intracranial Xenograft Model of Mouse

The U87-luc cell line was a gift from Dr. Shao-Chih Chiu (China Medical University, Taichung, Taiwan). It stably expresses a luciferase reporter gene derived from the U87MG GBM cell line. Nude mice of BALB/c (male, the National Laboratory Animal Center) at 2 months of age were anesthetized and fixation. A midline scalp incision was made and the skull was exposed. Next, a hole was drilled in the skull and cells (4 × 10^5^) suspended in medium (5 μL) were injected into the left striatum. Next, we healed wounds by bone wax and Dermabond skin adhesive (Ethicon Inc, Johnson and Johnson, Somerville, NJ, USA). Finally, for assessing tumor growth, we performed in vivo bioluminescence imaging analyses using the IVIS Lumina II system (Caliper Life Sciences, MA, USA). The median survival values of mice in the control and experimental groups were determined as described previously.

### 2.17. Luminescence Imaging of Intracranial Glioblastoma

Mice from Section 2.16 were randomly divided into a control group (saline only) (*n* = 3) and PMN-treated group (1 mg/kg, *n* = 3). The corresponding treatments (50 μL) were administered on days 10, 13 and 16 using intraperitoneal injection. Tumor size was measured every 10 days by biophotonic imaging quantification via the Xenogen IVIS 200 system (Xenogen, Palo Alto, CA, USA) starting from tumor implantation. Briefly, experimental mice were injected intraperitoneally (100-µL) with luciferase substrate D-luciferin (30-mg/mL, PerkinElmer) suspended in DPBS (Gibco). After 10 min, anesthesia was induced by inhalation of isoflurane gas with a nose cone. Images were recorded and quantified from the equivalent region of interest on the top of the mouse head using Living Image 4.1 software. Image intensity is shown as photon per square centimeter per second per steradian (ph/cm2/s/sr).

### 2.18. Statistical Analysis Methods

The experiment was repeated up to thrice. Statistical analysis of research data was performed using SAS software (SAS, Institute Inc., Cary, NC, USA). The data are shown as mean ± standard deviation (SD). We used one-way ANOVA and Tukey’s test to obtain assessments of statistical significance. The statistical significance was presented as *p* values < 0.05.

## 3. Results

### 3.1. Characterization of Alternatively Spliced Variants of the Hydroxysteroid 11-Beta Dehydrogenase 1 like Gene (HSD11B1L) in GBM

**Figure 1:**
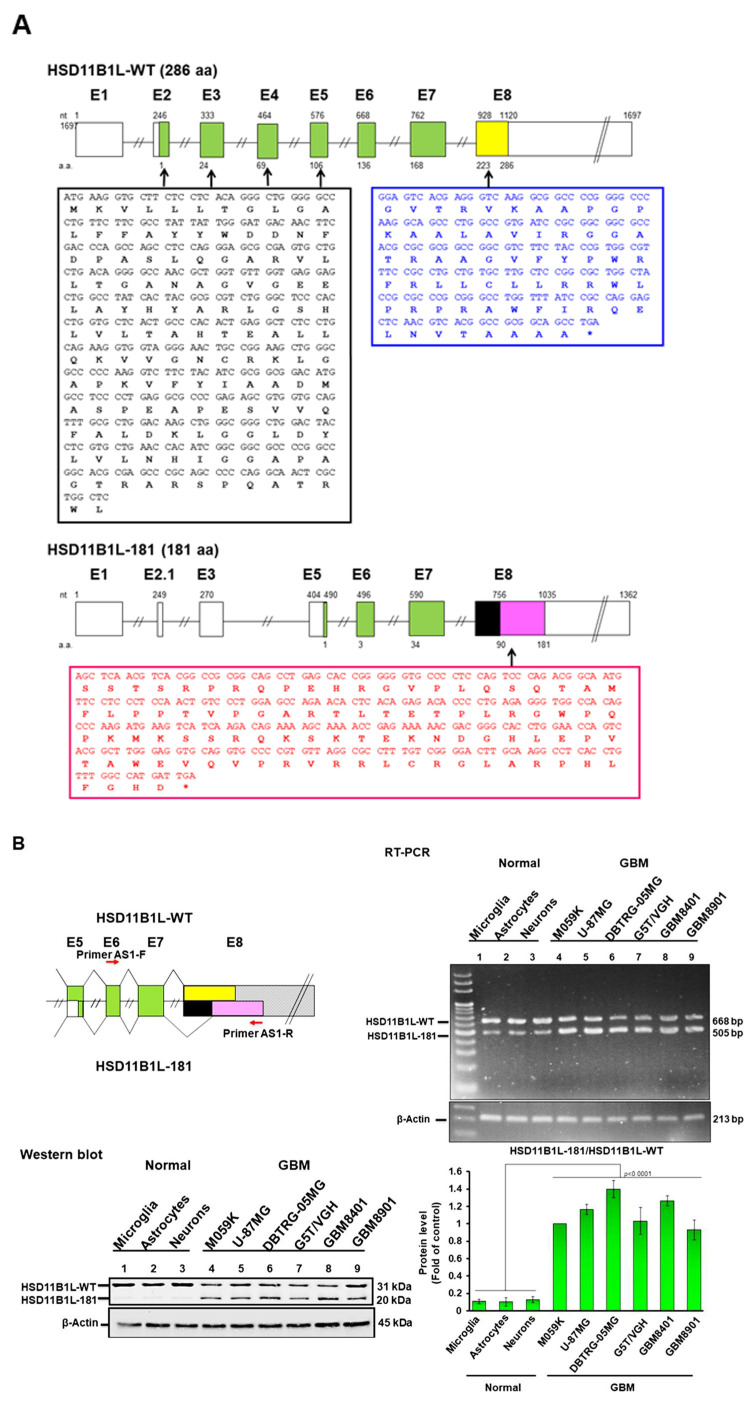

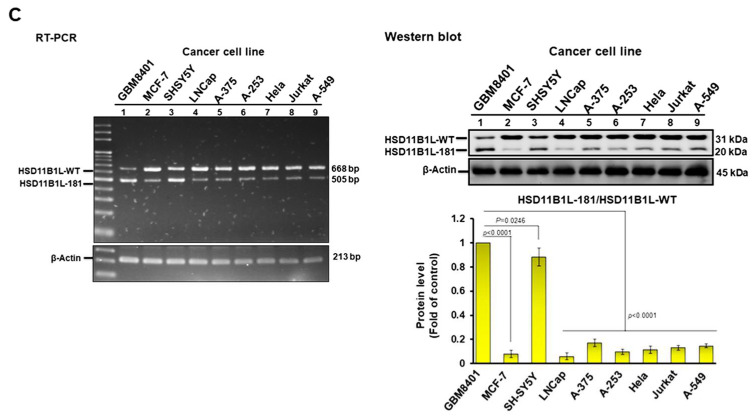
The splice variant HSD11B1L-181 of the HSD11B1L gene predominates in GBM. (**A**) Simplified transcript structure maps of HSD11B1L-WT and HSD11B1L-181. HSD11B1L-WT mature transcript consists of 8 exons. HSD11B1L-181 lacks exon 2 and exon 4, but adds to exon 2.1, causing the reading frame to shift to the penultimate amino acid at the end of exon 5 (a total of 134 amino acids are deleted, black box). Moreover, the splicing site changes in exon 8 resulted in partial deletion of the nucleotide sequence and shift of the reading frame. Finally, the original 63 amino acid sequence (blue box) was replaced with a different 92 amino acid sequence (red box). (**B**) Expression of HSD11B1L-181 is low in healthy human cells in brain but high in GBM cell lines. RT-PCR using designed primers on exon 6 and exon 8 to obtain HSD11B1L-WT and HSD11B1L-181 mature transcript fragments (top left). Representative results of agarose gel electrophoresis showing mature transcripts of HSD11B1L (668 bp) and HSD11B1L-181 (505 bp) (top right). Western blot showing bands for HSD11B1L-WT at 31 kDa and HSD11B1L-181 at 20 kDa (bottom left). β-actin is an internal loading control. Results were quantified using ImageJ (bottom right). (**C**) RT-PCR (top left) and Western blotting analysis (top right) showed that HSD11B1L-181 is less expressed in other cancer cell lines. The internal control is β-actin. Results were quantified using ImageJ (bottom right).

According to previous reports [18], we first established and amplified the cDNA library of GBM8401 cells. Next, according to the bioinformatics data of Ensembl (http://asia.ensembl.org/Homo_sapiens/Gene/Summary?g=ENSG00000167733;r=19:5680604-5688523) (accessed on 20 May 2022)., we designed a combination of primers for exon 1 and exon 8 of hydroxysteroid 11-beta dehydrogenase 1 like gene (*HSD11B1L*) to perform a *Taq* DNA polymerase-dependent PCR reaction. The product was then ligated to a carrier vector using TA cloning. Finally, the plasmids were transformed into *Escherichia coli* for replication and sequencing. We obtained two major transcripts present in GBM8401 cells. Nucleotide sequencing revealed that the larger transcript was wild-type HSD11B1L (HSD11B1L-WT, encoding 286 amino acids), while the smaller splice variant transcript was named HSD11B1L-181 (encoding 181 amino acids) (Figure 1A). Compared with HSD11B1L-WT, HSD11B1L-181 lacks exon 2 and exon 4, but adds to exon 2.1, causing the reading frame to shift to the penultimate amino acid at the end of exon 5 (a total of 134 amino acids are deleted, black box) (Figure 1A, black box). Moreover, the splicing site changes in exon 8 resulted in partial deletion of the nucleotide sequence and shift of the reading frame. Finally, the original 63 amino acid sequence (Figure 1A, blue box) was replaced with a different 92 amino acid sequence (Figure 1A, red box).

### 3.2. HSD11B1L-181 Mature Transcript Is Predominantly Expressed in GBM

Using the primer pair of AS1-F (in exon 6) and AS-1R (in exon 8) to perform RT-PCR (Figure 1B, top left) on the total RNA of cells can distinguish the mature transcript expression of HSD11B1L-WT (668 nt) and HSD11B1L-181 (505 nt); in addition, using HSD11B1L antibody (polyclone) can also distinguish the protein expression of HSD11B1L-WT (31kDa) and HSD11B1L -181 (20kDa). RT-PCR results showed that, compared with normal brain cells such as neurons, astrocytes and microglia, HSD11B1L-181 was highly expressed in GBM cell lines such as GBM8401, GBM8901, M059K, U-87MG, G5T/VGH and DBTRG-05MG (*p* < 0.0001, Figure 1B, top right). Similarly, in Western blotting, HSD11B1L-181 (20 kDa) was highly expressed in GBM cell lines compared with normal brain cells (Figure 1B, bottom left). Additionally, we also evaluated the level of HSD11B1L-181 in other cancerous cell lines. The results showed that the mature transcript (Figure 1C left, *p* < 0.0001) and protein (Figure 1C right, *p* < 0.0001) expressions of HSD11B1L-181 in the lung (A549), T lymphocytes (Jurkat), salivary gland (A-253), skin (A-375), cervix (HeLa), prostate (LNCap) and human breast cancer (MCF-7) were lower than those of the GBM8401 cell line. However, brain tumor SH-SY5Y cells had similar expression to GBM cell lines (Figure 1C). These results demonstrate that the alternative splice variant HSD11B1L-181 is highly expressed exclusively in human GBM cells.

### 3.3. HSD11B1L-181 but Not HSD11B1L-WT Can Promote GBM Cell Proliferation, Migration and Invasion

**Figure 2:**
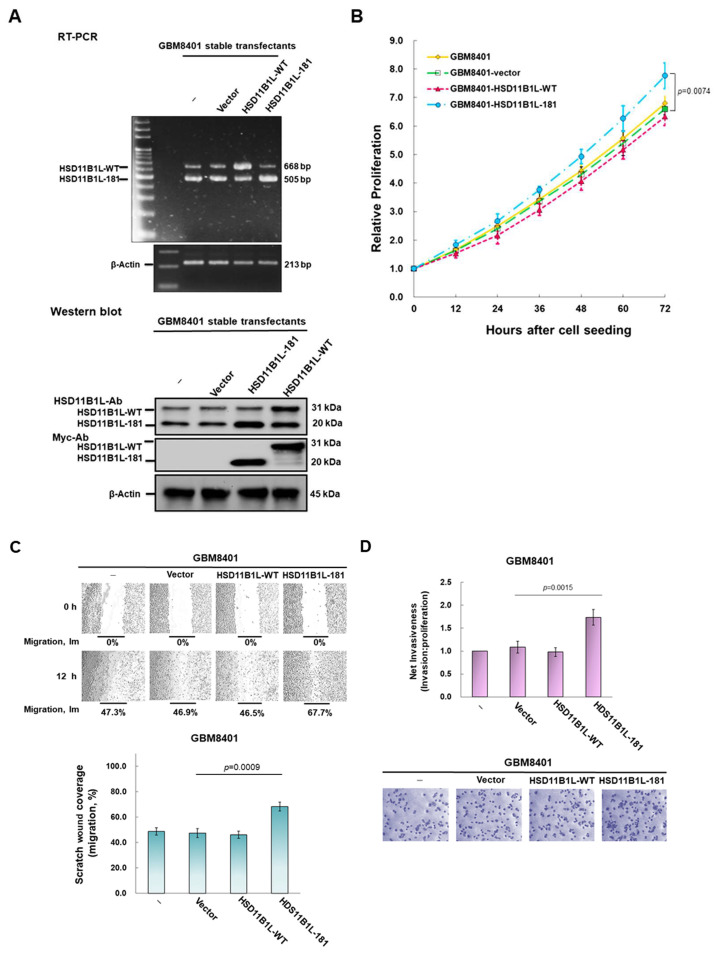
Overexpression of the splice variant HSD11B1L-181 enhances the proliferation, migration and invasive phenotypes of GBM8401 cells. (**A**) The expression of HSD11B1L-WT and HSD11B1L-181 in three stably transfected GBM8401 cell lines was analyzed by RT-PCR (top) and Western blotting (bottom). The internal loading control is β-actin. (**B**) The proliferation capacity of three stably transfected cell lines of GBM8401 was quantified using the CellTiter Blue Cell Viability assay. (**C**) Migration capacity of three stably transfected GBM8401 cells was quantified using a scratch assay (50×, top) and quantified results are presented in a bar graph (bottom). At the beginning of the experiment, the width of the scratch gap with the tip of the micropipette was taken as g0. The migration index Im is defined as Im = (gap t_0_ − gap t_12_)/gap t_0_, where gap t_12_ is the gap width after a time of 12 h. (**D**) The invasive properties of three stably transfected GBM8401 cells were quantified using the InnoCyte Cell Invasion Assay. Cells invading the basement membrane were stained by fluorescent Calcein-AM (top) or crystal violet (100×, bottom) to quantify 24 h after the assay was run.

To evaluate the role of HSD11B1L-181 in GBM, we established three GBM8401 stable transfecting lines with empty vector, HSD11B1L-WT and HSD11B1L-181, respectively. We analyzed the expression of HSD11B1L-WT and HSD11B1L-181 mature transcripts in GBM8401 cells using RT-PCR (Figure 2A, top) and Western blotting (Figure 2A, bottom). These results appear to be in line with our expectations.

To analyze the effect of HSD11B1L-181 on cell proliferation, we used the CellTiter Blue Cell Viability Assay Kit to track changes in cell number. The results showed that overexpression of HSD11B1L-181 significantly augmented the proliferation capacity of GBM8401 cells within 72 h. Cell proliferation was increased nearly 1.2-fold (*p* = 0.0074) compared with the control vector group (Figure 2B). In contrast, overexpression of HSD11B1L-WT had no effect on cell proliferation.

Next, we evaluated the effect of HSD11B1L-WT and HSD11B1L-181 on the migratory ability of GBM8401 cells using a scratch assay (Figure 2C, top). The results showed that the average Im values of the three stably transfected cell lines at t12 were 46.9% (empty vector), 46.5% (HSD11B1L-WT) and 67.7% (HSD11B1L-181) (Figure 2C, bottom). Overexpression of HSD11B1L-181 increased the migration ability of GBM8401 cells nearly 1.4-fold (*p* = 0.0009) compared with the empty vector (control) group. However, overexpression of HSD11B1L-WT did not affect cell migration (Figure 2C, bottom).

Furthermore, the effect of HSD11B1L-181 on the invasion ability of GBM8401 cells was assessed (Figure 2D) using fluorescent staining quantification of invaded cells (top) and staining of crystal violet (bottom). The metric assessment of net invasiveness is presented by calculating the invasion/proliferation ratio. The results showed that, compared with the control vector group, the invasion ability of GBM8401 cells overexpressing HSD11B1L-181 increased nearly 1.6 fold (*p* = 0.0015). However, overexpression of HSD11B1L-WT did not alter cell invasive properties. These experimental results indicate that HSD11B1L-181 can promote the proliferation, migration and invasion of GBM cells.

### 3.4. Downregulation of the Expression of HSD11B1L-181 via RNAi Suppress Characteristics of Proliferation, Migration and Invasion of GBM Cells

**Figure 3:**
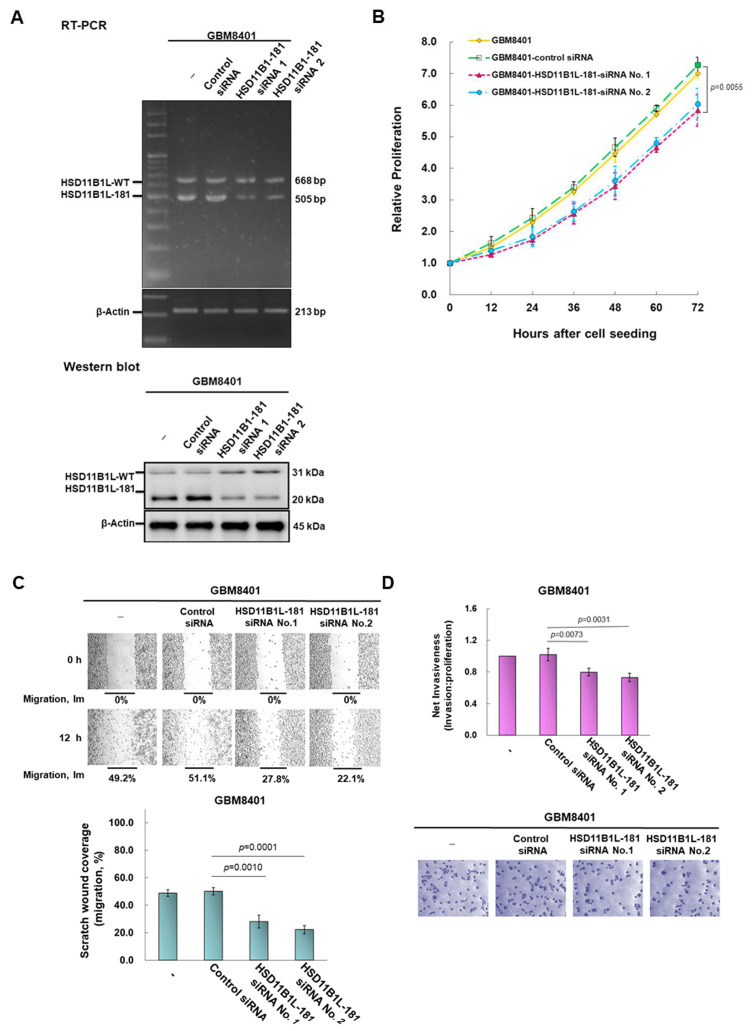
Down-regulating the expression of HSD11B1L-181 by siRNA can obviously reduce the proliferation, migration and invasion abilities of GBM 8401 cells. HSD11B1L-181 siRNA was transfected into GBM8401 cells and cultured for 24 h. (**A**) HSD11B1L-WT and HSD11B1L-181 expression was confirmed by RT-PCR (top) and Western blotting (bottom). The internal loading control is β-actin. (**B**) The proliferation capacity of GBM8401 cells in each group was quantified using the CellTiter Blue Cell Viability Assay. (**C**) The migratory ability of GBM8401 cells in each group was shown using a scratch test (50×, top). Bar graphs represent quantified experimental results (bottom). (**D**) The invasive properties of GBM8401 cells in each group were determined using the InnoCyte cell invasion assay. Cells invading the basement membrane were fluorescently stained and quantified (top) or cell bodies were stained with crystal violet (100×, bottom) 24 h after the experiment.

We designed a siRNA targeting HSD11B1L-181 against exon 2.1. RT-PCR and Western blotting analysis showed that the expression of HSD11B1L-181 in GBM cells was significantly decreased after HSD11B1L-181 siRNA treatment (Figure 3A). Western blotting showed a 70% reduction in HSD11B1L-181 protein expression (No. 1, *p* < 0.0001). Such conditions resulted in a 20% reduction in the proliferation capacity of GBM8401 cells compared to the control siRNA group (No. 1, *p* = 0.0055) (Figure 3B). Furthermore, the migratory and invasion abilities of the cells were reduced by 44% (No. 1, *p* = 0.0010) (Figure 3C) and 22% (No. 1, *p* = 0.0073) (Figure 3D) compared to the control siRNA group, respectively.

### 3.5. Specific Interaction between HSD11B1L-181 and Parkin

**Figure 4:**
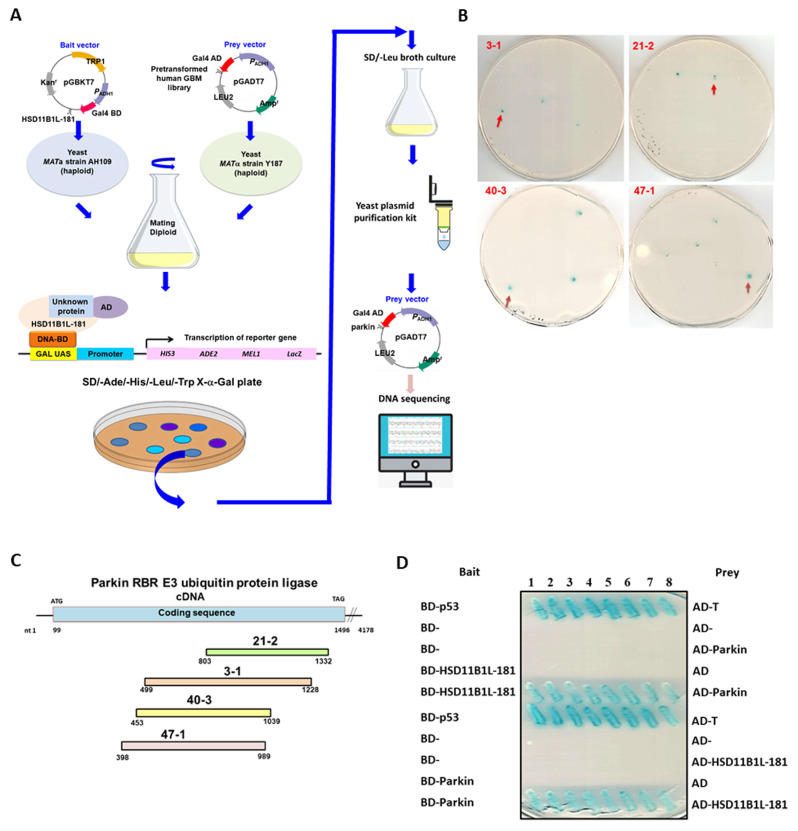

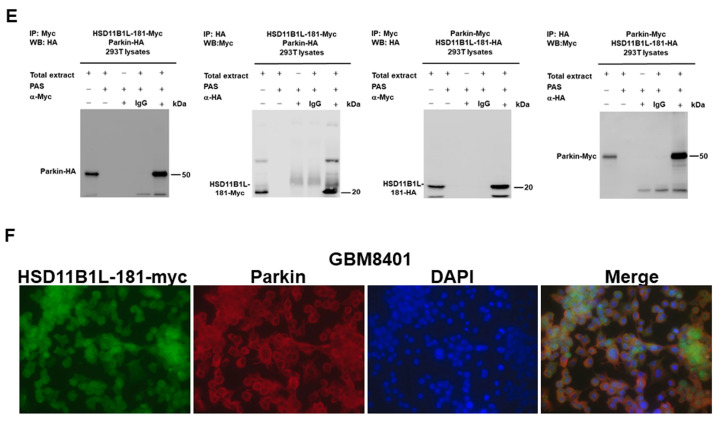
The specific interaction between HSD11B1L-181 and parkin (PARK2) in yeast and cells. (**A**) Schematic diagram of yeast two-hybrid screening using HSD11B1L-181 as bait. Screening of parkin prey clones from an GBM8401 cDNA library. Plasmids of HSD11B1L-181 with a Gal4 DNA-binding domain (BD) fusion were transformed into the AH109 strain. It was then mated with the Y187 strain containing plasmids for an GBM8401 cDNA library fused to the Gal4 activation domain (AD). If the interaction between the fusion proteins expressed by the two exists, the reporter gene of diploid cells can be activated and the blue clone can be formed on the SD/-Ade/-His/-Leu/-Trp/X-α-gal plate. (**B**) Positive blue diploid colonies showing parkin cDNA fragments. (**C**) Schematic representation of the sequencing of four colonies with parkin cDNA fragments (NCBI reference sequence: NM_004562). (**D**) The interaction of HSD11B1L-181 with the coding region of the full-length parkin cDNA was confirmed by yeast two-hybrid analysis. The interaction of BD-p53 and AD-T in diploid cells served as a positive control group. (**E**) Interaction of HSD11B1L-181 with parkin was confirmed using immunoprecipitation analysis of 293T cells expressing HSD11B1L-181-Myc and parkin-HA. Reverse immunoprecipitation was done simultaneously. (**F**) Using immunofluorescent staining, it was revealed that part of HSD11B1L-181 (green fluorescence) and part of parkin (red fluorescence) were co-localized in GBM8401 cells (400×). DAPI (blue fluorescence) was used to visualize nuclei.

To further explore the possible mechanisms by which HSD11B1L-181 promotes the carcinogenesis potential of GBM, we identified its possible interacting target proteins by yeast two-hybrid screening. First, we transformed plasmid with Gal4 DNA-binding domain (Gal4-BD) in-frame fusion HSD11B1L-181 coding DNA into AH109 strain (type a) as a bait. A cDNA expression library of GBM8401 in yeast Y187 strain (α type) was screened. Some 7.1 × 10^5^ colonies were obtained by mating yeast for screening. Finally, 34 positive clones were obtained (Figure 4A). Isolation and sequencing of the prey plasmid within the positive clones revealed that four clones contained part of the cDNA coding region of parkin (PARK2) (Figure 4B). The largest positive clone (3–1) was 730 bp in length, including the coding region at the C-terminal part of parkin (Figure 4C). Parkin is related to mitophagy [22], apoptosis and tumorigenesis [23].

Next, we transformed the pGBKT7 vector containing intact coding DNA sequence of HSD11B1L-WT or HSD11B1L-181 and the pGADT7 vector containing intact coding DNA sequence of parkin into AH109 and Y187 strains of yeast, respectively. Then, the yeast two-hybrid test was performed. The results showed that HSD11B1L-181 specifically interacted with parkin (Figure 4D), but not with HSD11B1L-WT. Additionally, we co-transfected the plasmid expressing HSD11B1L-181-Myc and parkin-HA (or the plasmid expressing parkin-Myc and HSD11B1L-181-HA) into 293T cells. Cell extracts were prepared for immunoprecipitation analysis. The results showed that HSD11B1L-181 and parkin were present on a co-immunoprecipitated complex (Figure 4E). Finally, immunofluorescence staining showed that part of HSD11B1L-181-myc (green fluorescence) co-localized with part of parkin (red fluorescence) in the cytoplasm (Figure 4F). These results show a direct and specific cytoplasmic interaction of HSD11B1L-181 with parkin.

### 3.6. HSD11B1L-181 Uses the C-Terminal Amino Acid Fragment to Interact with the Middle Amino Acid Fragment of Parkin

**Figure 5:**
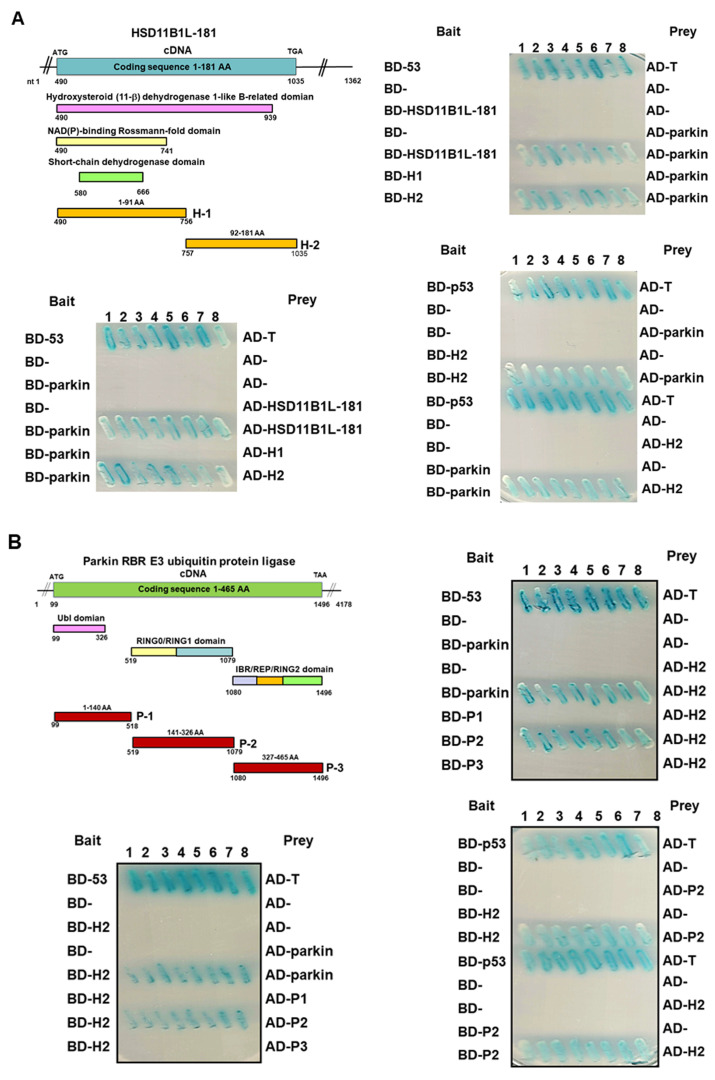
HSD11B1L-181 interacts mainly through the segment of C-terminal and the middle region of parkin using an assay of yeast two-hybrid. (**A**) Schematic representation of the two HSD11B1L-181 deletion mutants used in this assay (top left). One is the amino acid sequence 1–91 (H1 fragment) and the other is the amino acid sequence 92–181 (H2 fragment). The results showed that only the H2 fragment specifically interacted with parkin. (**B**) Schematic representation of the three parkin deletion mutants used in this assay (top left). One is the amino acid sequence 1–140 (P1 fragment), the second is the amino acid sequence 141–326 (P2 fragment) and the last is the amino acid sequence 327–465 (P3 fragment). The results showed that only the P2 fragment of parkin specifically interacted with the H2 fragment of HSD11B1L-181 (top right, bottom left and bottom right). Diploid cells expressing BD-p53 and AD-T were used as positive controls.

Further, we wanted to clarify the possible region of interaction between HSD11B1L-181 and parkin. We constructed two deletion mutants of the cDNA sequence in the coding region of HSD11B1L-181. These are H1 (fragment comprising amino acids 1–91) and H2 (fragment comprising amino acids 92–181), respectively. The sequence for H2 was absent from HSD11B1L-WT (Figure 5A top left). We determined the interaction of the HSD11B1L-181 deletion mutant with parkin using the yeast two-hybrid assay. The results showed that deletion of the H2 region (92–181 fragment) from HSD11B1L-181 disrupted the interaction with parkin (Figure 5A top right, bottom left and bottom right). Therefore, the H2 domain of HSD11B1L-181 is necessary for the interaction with parkin. Additionally, we separated parkin into three parts, namely, P1 (containing 1–140 amino acids), P2 (containing 141–326 amino acids) and P3 (containing 327–465 amino acids). Then, the H2 region of HSD11B1L-181 was used as bait to confirm the possible interaction region on parkin. The results showed that only the P2 fragment could interact with the H2 region (Figure 5B). Since the P2 region of parkin is related to its activity, HSD11B1L-181 may specifically block the function of parkin and inhibit its anti-cancer ability, thus enhancing the carcinogenesis potential of GBM.

### 3.7. The Proliferation, Migration and Invasion Ability of GBM Cells Will Be Significantly Augmented by Parkin Knockdown

**Figure 6:**
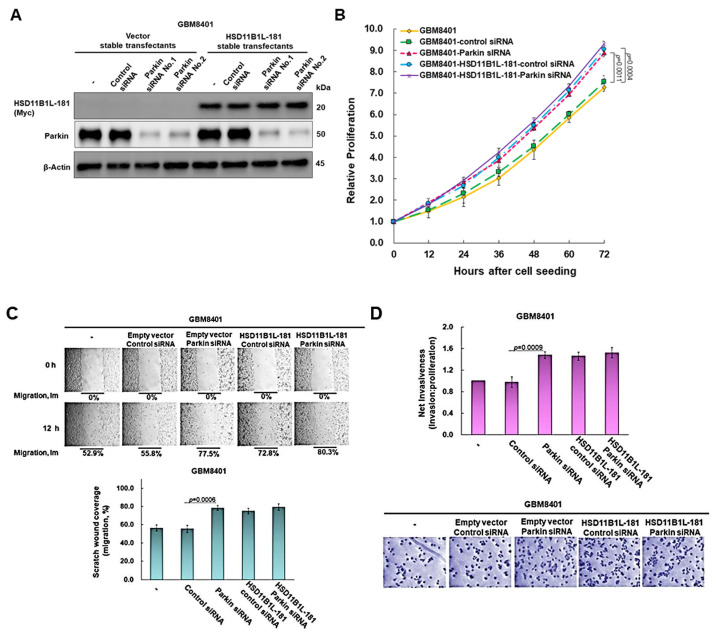
Overexpression of HSD11B1L-181 enhanced the proliferation, migration and invasion properties of GBM cells in part by inhibiting the activity of parkin. GBM8401 cells in the control vector group or HSD11B1L-181 overexpression group were delivered parkin-specific or control non-specific siRNA. After 24 h of culture, the cell proliferation, migration and invasion abilities were analyzed. (**A**) The expression of HSD11B1L-181-myc and parkin was detected using Western blot. β-actin is the internal loading control. (**B**) Cell proliferation capacity was measured via the CellTiter Blue Cell Viability assay. (**C**) Scratch test to determine cell migration ability (top). The bar graph presents quantitative data (bottom). (**D**) Cell invasion ability was determined by a transwell invasion assay. Cells invading the basement membrane were fluorescently stained and quantified 24 h after the experiment (top) and finally confirmed with crystal violet staining (bottom).

To clarify the effect of parkin on proliferation, migration and invasion in GBM, we used siRNA to knock down the expression of parkin in control vector-transfected GBM8401 cells. After treating 24 h, in the parkin siRNA group, the expression of parkin was reduced by 90% compared with the control siRNA group (No. 1, *p* < 0.0001) (Figure 6A). Further studies found that the knockdown of parkin significantly enhanced the proliferation (*p* = 0.0011, Figure 6B), migration (*p* = 0.0006, Figure 6C) and invasion (*p* = 0.0009, Figure 6D) abilities of GBM8401 cells.

### 3.8. Parkin Knockdown Does Not Enhance the Ability of HSD11B1L-181 to Promote the Proliferation, Migration and Invasion of GBM Cells

We further analyzed the role of parkin in the enhancement of GBM carcinogenesis by HSD11B1L-181. Using siRNA for parkin reduced parkin expression by 93% in the HSD11B1L-181 overexpression group (No. 2, *p* < 0.0001) (Figure 6A). The data revealed that HSD11B1L-181 overexpression coincided with parkin downregulation without significant cumulative enhancement of the carcinogenesis properties of GBM (Figure 6B–D). According to the above results, we speculate that the tumor-promoting potential of HSD11B1L-181 may be achieved partly by binding and inhibiting the activity of parkin, and finally enhancing the characteristics of proliferation, migration and invasion of GBM cells.

### 3.9. PMN Can Interfere with the Interaction between HSD11B1L-181 and Parkin in the Yeast Two-Hybrid Model

**Figure 7:**
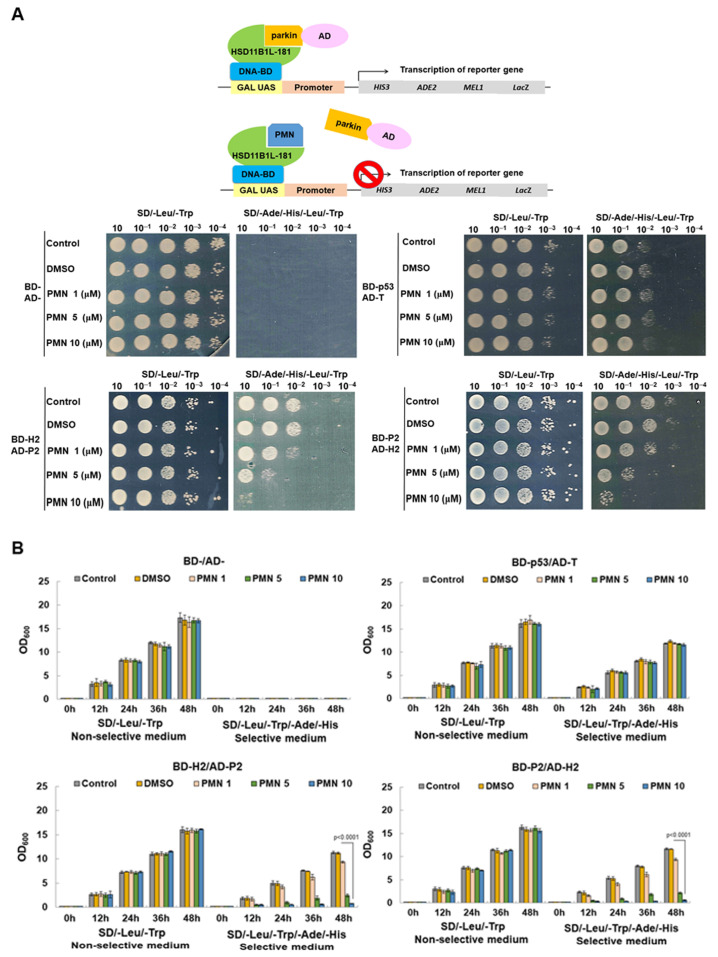
The interaction of HSD11B1L-181 with parkin is inhibited by PMN in a yeast two-hybrid model. (**A**) Schematic presenting the strategy for screening inhibitors of HSD11B1L-181 interaction with parkin using a yeast two-hybrid-based growth assay (top). Log-phase cultures of diploid cells expressing the indicated fusion proteins of BD and AD were spotted in the non-selective (-Leu-Trp) and selective (-Leu-Trp-Ade- His) plates including different PMN concentration and incubated at 30 °C for 3 days. (**B**) The indicated diploids, grown in nonselective media, were inoculated into selective and nonselective liquid media containing series dilutions of PMN. Diploid cells were diluted to OD_600_ = 0.2 as the starting point and the OD was measured every 12 h for a total of 48 h. The bar graph shows the mean determination of OD_600_ values from three experiments.

Next, we wanted to establish possible interference strategies for interacting HSD11B1L-181 with parkin. The simplest strategy is to screen for the candidate compounds that block this interaction. We screened several phytocompounds in our laboratory using a yeast two-hybrid-based growth assay (Figure 7A and Figure S1). The results of the study show that PMN has this effect. On SD/-Leu/-Trp plates, yeast spot assays showed that diploid cells with different combinations of expression vectors grew normally when PMN treatment was below 10 mM (Figure 7A). This indicates that doses of PMN below 10 mM are not toxic to yeast. In contrast, on SD/-Ade/-Leu/-His/-Trp plates, BD-H2 (HSD11B1L-181)/AD-P2 (parkin) and BD-P2/AD- H2 diploid cells showed a dose-dependent growth inhibition of PMN (Figure 7A). The growth of BD-P53/AD-T diploid cells on SD/-Ade/-Leu/-His/-Trp plates were not affected (Figure 7A). For accurate quantification, we used OD_600_ to measure the amount of yeast. These data appear similar to those of the yeast spot assay (Figure 7B). After 48 h of culture, the growth rate of the 10 mM PMN group was reduced by 93% (*p* < 0.0001, Figure 7B bottom left) and 95% (*p* < 0.0001, Figure 7B bottom right) in BD-H2/AD-P2 and BD-P2/AD- H2 groups compared with the DMSO group, respectively. The growth of diploid cells expressing BD-P53 and AD-T was unchanged (Figure 7B). This indicated that PMN treatment could effectively block the interaction between HSD11B1L-181 and parkin.

### 3.10. PMN Treatment Inhibits the Proliferation, Migration and Invasion of GBM Cells in Vitro

**Figure 8:**
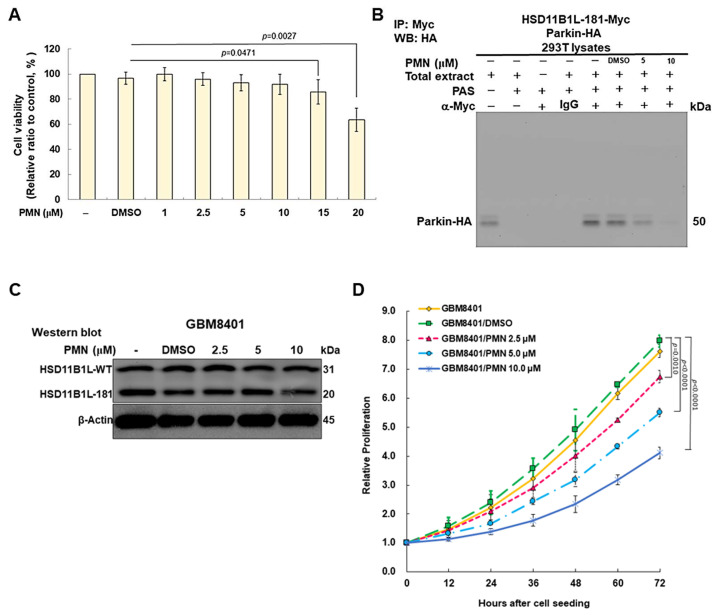

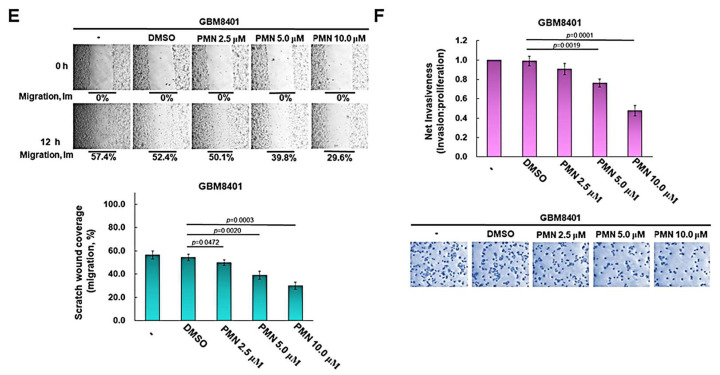
The proliferation, migration and invasion abilities of GBM cells were reduced by PMN treatment. (**A**) TUNEL assay was used to evaluate the cell viability of GBM8401 cells treated with different concentrations of PMN after 24 h. Results showed that cell survival was not affected at treatment concentrations below 10 μM PMN. (**B**) PMN inhibits the interaction of HSD11B1L-181 and parkin in a co-immunoprecipitation assay. 293T cells expressing HSD11B1L-181-myc and parkin-HA were lysed, then 5 or 10 μM PMN added, and reacted with anti-myc antibody overnight. Finally, parkin was identified on the co-immunoprecipitated complex by Western blotting with an anti-HA antibody. (**C**) Western blotting of the expression of HSD11B1L-WT and HSD11B1L-181. β-actin was the control for internal loading. (**D**) Using CellTiter Blue Cell Viability assay to analyze the effect of PMN treatment on the proliferation of GBM8401 cells. (**E**) The effect of PMN treatment on the migration of GBM8401 cells was analyzed using a scratch assay (50×, top). The bar graph shows the statistical results of the scratch assay (bottom). (**F**) The effect of PMN treatment on the invasion of GBM8401 cells was analyzed by transwell assay. The invasive cells were quantified by cytofluorescent staining (top) on the basement membrane and confirmed with crystal violet staining (100×, bottom) after cells were incubated for 24 h.

The above experimental results showed that the interaction between HSD11B1L-181 and parkin in the yeast model could be effectively reduced by PMN treatment. Therefore, we further evaluated whether PMN could diminish the proliferation, migration and invasion of GBM cells in vitro. First, we use TUNEL Assay to determine the concentration of PMN treatment. Data indicated that the apoptotic rate of GBM8401 cells did not increase significantly when the PMN treatment concentration was less than 10 μM (Figure 8A). We therefore treated GBM with up to 10 μM PMN and assessed its utility in terms of tumor suppressive properties. Additionally, PMN dose-dependently decreasing the interaction between HSD11B1L-181 and parkin in 293T cell lysates was also shown by co-immunoprecipitation (Figure 8B). Furthermore, we confirmed by Western blotting that PMNs did not affect the expression of HSD11B1L-181 (Figure 8C). Next, we analyzed cell proliferation capacity. The results indicated that the proliferation of GBM8401 cells diminished dependently with the dose of PMN. The GBM8401 cell proliferation was decreased by 48% (*p* < 0.0001) with 10 μM PMN treatment (Figure 8D). The migration and invasion of GBM8401 cells also revealed a dose-dependent lessening of PMN. The cell migration and invasion abilities of GBM8401 cells were diminished by 45% (*p* = 0.0003) (Figure 8E) and 52% (*p* = 0.0001) (Figure 8F), respectively, under the treatment condition of 10 μM PMN.

### 3.11. PMN Treatment Prevents Tumor Growth in a Nude Mice Model of Subcutaneous Xenograft of GBM

**Figure 9:**
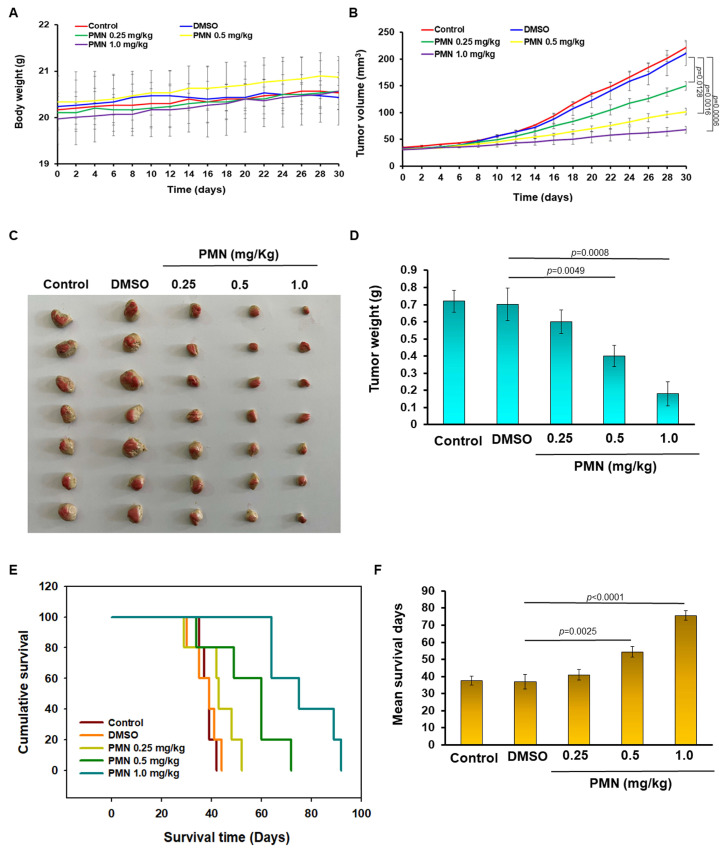
PMN treatment inhibits tumor growth in a nude mice model of dorsal subcutaneous GBM xenografts. The dorsal side of male nude mice were inoculated subcutaneously with GBM8401 cells. After tumor formation, drug injection and tumor growth analysis were performed. Mice received intraperitoneal injections of saline, DMSO, 0.25 mg/kg PMN, 0.5 mg/kg PMN and 1.0 mg/kg PMN, respectively, on days 4, 8, 12 and 16 after tumor development (*n* = 6). (**A**) The weight of mouse body was continuously recorded every 2 days during the experiment. The results showed that there was no significant change in the average body weight of the mice in each group. (**B**) During the experiment, the tumor volume of mice in each group was measured every 2 days to obtain tumor growth curves. The results showed a dose-dependent slowdown of tumor growth in PMN-treated mice compared with the control group. (**C**) On day 30 of the experiment, mice were sacrificed and tumors were excised for analysis. The results indicated that PMN treatment dose-dependently diminished tumor size in mice. (**D**) In tumor-bearing mice, the weight of tumor was significantly lighter in the PMN group compared with the DMSO group. (**E**) Evaluation of the median survival of mice in each group. (**F**) The median survival day of PMN-treated tumor-bearing mice was dose-dependently prolonged compared to the DMSO group.

We further used a nude mice model of dorsal subcutaneous GBM xenograft to evaluate the potential clinical application of PMN against GBM. According to the study by Yu et al., a therapeutic dose of PMN below 1.0 mg/kg had no obvious toxic effect on mice [24]. Therefore, we considered 1.0 mg/kg PMN treatment as the highest dose. Furthermore, the body weight of mice was not significantly affected during PMN treatment (Figure 9A). Our study showed that GBM8401 tumors in tumor-bearing mice were treated with 1.0 mg/kg PMN for 30 days and the tumor size was lessened by 70% compared with the control group (*p* = 0.0006) (Figure 9B). At the end of the experiment, mice subcutaneous tumors were excised and measured. The results showed a dose-dependent decrease in tumor size in the PMN group compared with the control group (Figure 9C). Treatment of tumor-bearing mice with PMN at 1.0 mg/kg decreased tumor weight by 74% (*p* = 0.0008) (Figure 9D). Finally, the median survival days of GBM mice was dose-dependently prolonged under PMN treatment compared with the control group (Figure 9E). In the group treated with 1.0 mg/kg PMN (75.7 ± 2.8 days) compared with the control group (37.0 ± 4.3 days), the mean extension was 38.7 days (Figure 9F).

### 3.12. PMN Treatment Inhibits Tumor Growth in Orthotopic GBM Xenograft Immunodeficient Mice

**Figure 10:**
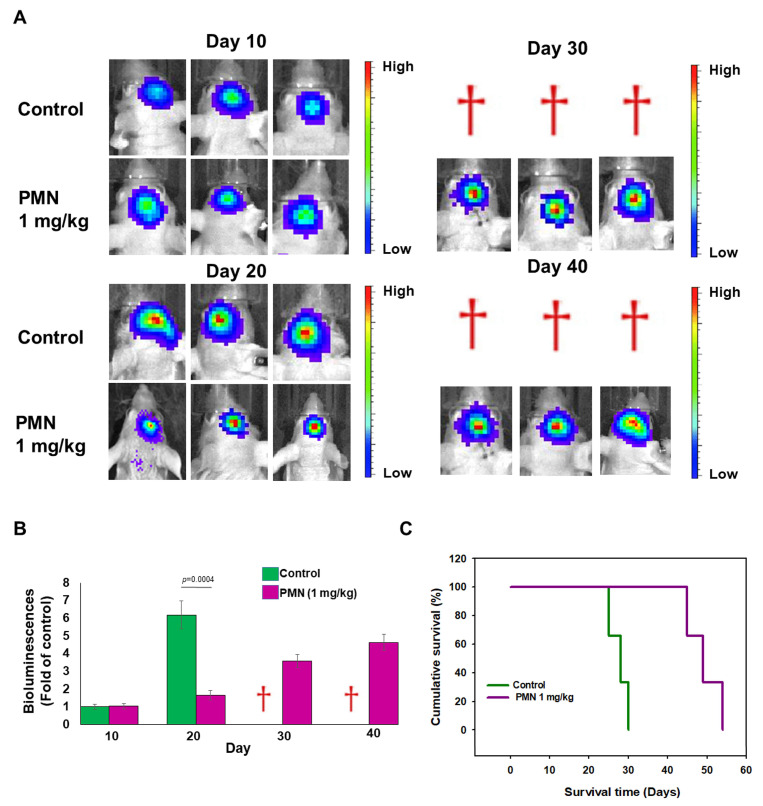
Biophotonic measurements demonstrate the antitumor efficacy of PMN treatment in immunodeficient mice with orthotopic GBM xenografts. U87-luc GBM cells (4 × 10^5^) were injected intracranially into male BALB/c nude mice (on day 0). Mice were injected intraperitoneally with saline (control group, *n* = 3) or 1.0 mg/kg PMN (*n* = 3) on day 10, day 13 and day 16, respectively. (**A**) Bioluminescence imaging of the cranium of surviving mice at days 10, 20, 30 and 40 after GBM cell implantation. Tumor burden is displayed by a colorimetric scale (function of total irradiance per steradian/s/cm^2^). The death of mice is represented by a red dagger. (**B**) Comparison of the results for the mean irradiance values for each group in panel A. (**C**) Kaplan—Meier curves of the control and PMN-treated groups show that PMN treatment effectively prolongs the average lifespan of GBM mice.

Next, we assessed the effect of PMNs in preventing the growth of orthotopic glioblastoma. We injected U87-luc cells into nude mice intracranially. Bioluminescence imaging was then used to track tumor growth. The results are shown in Figure 10A. Single bioluminescence images of the GBM of surviving mice in the control and experimental groups (1.0 mg/kg PMN) were obtained every 10 days until 40 days after injection of U87-luc cells. The results revealed that most mice developed similar small GBMs before treatment (day 10, Figure 10B). On day 20, the tumor size of mice treated with 1.0 mg/kg PMN was significantly decreased by 73% compared with the control group (*p* = 0.0004) (Figure 10B). By day 30, the control mice all died from the aggressive growth of GBM tumors. However, PMN-treated mice survived 100% with a slight tumor enlargement (Figure 10B). At day 40, PMN-treated mice were still alive (Figure 10B). Finally, the average days survived in the treated group was 48.3 days, compared with 26.7 days in the DMSO-treated group. PMN treatment prolonged the survival of GBM mice by 21.6 days (Figure 10C).

## 4. Discussion

Alternative splicing (AS) events of specific genes in cancer cells can reflect the tumor formation and the degree of progression. Correction of aberrant AS or its effects is an ongoing development in cancer treatment strategies [4]. In this study, we found that the AS variant HSD11B1L-181 of the *HSD11B1L* gene was highly expressed exclusively in GBM cell lines. However, its expression is low in healthy cells of the brain and other types of cancerous cells. Compared with HSD11B1L, the HSD11B1L-181 transcript lacks 134 amino acids at the N-terminus and the original 63 amino acids at the C-terminus are replaced by 92 different amino acid sequences. Additionally, when we overexpressed HSD11B1L-181 in GBM cells, it can significantly enhance the proliferation, metastasis and invasion characteristics of tumor cells. In contrast, inhibition of HSD11B1L-181 expression by siRNA significantly lessened the cancerous potential of GBM cells. Therefore, we confirmed that the generation of the HSD11B1L-181 splice variant is closely related to the malignant degree of GBM.

HSD11B1L is found on chromosome 19p13.3 of humans and is a member of the large short-chain dehydrogenase/reductase (SDR) family. SDR enzymes have various substrates, including steroids, xenobiotics and aromatic compounds. It has two conserved motifs, one for the nucleotide cofactor (NAD/NADP) binding domain and one for the catalytic active site domain. HSD11B1L is expressed mainly in the cytoplasm and endoplasmic reticulum of the brain. Its true reactive substrate remains unclear. Speculated function may be the regulation of tissue availability of physiologically relevant glucocorticoids [19]. Interestingly, it is completely absent in all rodent and rabbit genomes. HSD11B1L is predominantly expressed in the anterior pituitary and ovarian granulosa cells by analysis of nonhuman primate marmosets and sheep [19].

To explore the possible mechanism of HSD11B1L-181 to enhance the oncogenesis of GBM, we employed cDNA expression library screening by yeast two-hybrid to identify its candidate interacting factors. The results indicated that parkin (PARK2) is one of the interaction partners. The main function of parkin is E3 ubiquitin ligase, which is involved in the degradation pathway of specific ubiquitin-labeled proteins to proteasomes or lysosomes [25]. Additionally, when the mitochondria of cells are abnormal, parkin can recognize the specific protein PINK1 on the outer membrane and clear them by promoting mitophagy [26]. Furthermore, parkin can regulate apoptosis through mitochondria-dependent and -independent pathways [27]. Thus, mutations in parkin are linked with the death of dopamine neurons in Parkinson’s disease [26] and in various malignancies including GBM [28].

Chromosome 6q (Ch6q) is a hotspot of genomic alterations in GBM, often deleted or hypermethylated. Parkin, located within this region, has been designated as a tumor suppressor for GBM [29]. TP53 is a suppressor gene of tumor that can regulate the cell cycle to induce apoptosis or cell senescence and maintain the stability of the genome, and its mutation leading to its inactivation is considered as a key etiological factor in the development of various cancers. Studies have revealed that parkin transcription is regulated by p53. Loss of function of p53 in GBM observed downregulation of parkin expression [30]. Studies have revealed that the overexpression of parkin can decrease the proliferation of GBM cells, and its expression level is positively correlated with the survival outcome of GBM patients but negatively correlated with brain tumor grades of different cell origins [31].

G1/S cyclins, such as Cyclin D and E, are one of the master regulators in cancer. Accumulation of the cyclin D-cyclin-dependent protein kinase (CDK)4/6 complex partially phosphorylates the retinoblastoma tumor suppressor protein (Rb), whose inhibition is important for cell cycle progression [32]. The cyclin E-CDK2 complex phosphorylates the cyclin D inhibitor p27Kip1, marks it for degradation, initiates the assembly of the pre-replication complex and promotes the expression of cyclin A, which determines the initiation of DNA replication [33]. Studies have shown that parkin can be regarded as a GBM inhibitor because its overexpression prevents cells from entering the S phase by promoting ubiquitination and degradation of cyclins D and E [34]. The cyclin A-CDK2 complex is required for cell entry into the S phase, displaces cyclin E and initiates DNA replication. The M phase requires the interaction with cyclin A-CDK1. Cyclin B-CDK1 is required for cells to enter and exit the M phase of the cell cycle [35]. Rouland et al. indicated that parkin expression was lessened in GBM specimens and that its expression was inversely correlated with that of cyclins A and B [28]. Parkin prevents the proliferation of GBM cells by regulating the S phase and G2/M phase of the cell cycle by trans-repressing the expression of cyclin A and cyclin B genes. Parkin inactivation leads to enhanced tumor progression in mouse models [28].

Additionally, studies have shown that the expression of hypoxia-inducible factor (HIF-1α) and metastasis in breast cancer are negatively correlated with parkin expression. HIF-1α ubiquitination and degradation via parkin abolished breast tumor metastasis [36]. Regional hypoxia is also characteristic of GBM. Parkin negatively regulates the expression of HIF-1α and HIF-3α in GBM under hypoxic conditions, resulting in reduced expression of pro-angiogenic factors and tumor invasiveness [37]. Studies have shown that the malignant progression of GBM is related to the overexpression and hyperactivation of EGFR. It enhances GBM ability to proliferation and invasive and resist apoptosis [38]. The EGFR-specific E3 ligase is parkin. Parkin increases EGFR ubiquitination and inhibits GBM [39]. Moreover, parkin ubiquitinates pyruvate kinase M2 (PKM2). The stability of PKM2 is not affected by ubiquitination, but the enzyme activity will be reduced to affect the glycolytic pathway and cell metabolism, finally inhibiting the growth of GBM [40].

Further, we knocked down parkin using siRNA and found that it promoted GBM cell proliferation, metastasis and invasion as previously reported. Our results also indicated that the strong interaction between the two proteins is mainly through the C-terminal segment (92–181) of HSD11B1L-181 and the middle fragment (141–326) of parkin. Parkin includes Ub-like domain (Ubl), RING finger (RING0 and RING1) domain, in-between-RING (IBR), repressor (REP) region and RING2 domain. The middle fragment of parkin contains RING0/RING1 domains. RING1 develop the binding site for E2 Ub-conjugating enzyme. Its mutation or deletion may block its binding site with E2 Ub-conjugating enzyme, thus abolishing the activity of its own E3 ubiquitin ligase, resulting in the functional defect of parkin [41]. Therefore, we speculate that HSD11B1L-181 may enhance the carcinogenesis potential of GBM by abolishing parkin function on cell cycle factors and HIF1 by interacting with the middle region of parkin.

On the treatment strategies derived from this study, we found that PMN can effectively prevent the interaction between HSD11B1L-181 and parkin by using yeast two-hybrid based growth assay and co-immunoprecipitation. The alkaloid PMN derives from the bulbs of *Fritillaria thunbergii* Miq, and has several pharmacologic properties linked to anti-inflammatory [42], anti-cancer [24,43] and antioxidant activity [44]. Therefore, in addition to the ability to hinder the interaction between HSD11B1L-181 and parkin, PMN also blocks the activation of inflammatory factors and cytokines [45]. Likewise, it reduces intracellular ROS production [44]. Abnormal NF-κB activity [46] and cellular ROS level [47] are associated with GBM progression and malignancy. Furthermore, high-dose PMN treatment can stop the cell cycle by inhibition of the Akt/GSk3β signaling pathway and blocks autophagic flux by arresting AMPK-ULK1 axis to prevent the growth of GBM in cell and mouse models [48]. According to the above, PMN possesses multiple tumor suppressor properties that may effectively deepen the therapeutic utility for GBM.

Finally, we wanted to ask how is the AS variant of HSD11B1L-181 produced in GBM cells? In the process of cell cancerization, there are two main reasons for the formation of the abnormal AS event. The first is mutations, deletions, insertions or modifications of cis-regulatory elements in the gene sequence, thus altering AS sites. However, searching the Cancer Genome Atlas Glioblastoma Multiforme (TCGA-GBM) data did not find any significant specific mutations in the HSD11B1L gene sequence. The second is the abnormal expression or mutation of transactivating splicing factors (TSF), which changes the TSF level, activity and location in cells, and finally leads to aberrant AS. About four hundred splicing factors mediate GBM-associated AS [18]. Fuentes-Fayos et al. found that, compared with healthy brain samples, the expression of spliceosome components and splicing factors of SRSF3, RBM22, PTBP1 and RBM3 in GBM are seriously dysregulated. Silencing of these factors reduces the in vitro invasiveness parameters and induces apoptosis, particularly SRSF3 [49]. Huang et al. found that the abnormal splicing factor CUGBP Elav-like family member 5 (CELF5) significantly regulates the AS of Germ Cell-Specific Gene 1-Like Protein (GSG1L), which may be central to the occurrence and prognosis of GBM [50]. Li et al. confirmed that m^6^A methyltransferase METTL3 sustained its oncogenic role by modulating nonsense-mediated mRNA decay (NMD) of serine- and arginine-rich splicing factors (SRSF) and AS isoform switches in GBM [51]. We analyzed the sequence of the *HSD11B1L* gene using RBPmap (Version 1.2) [52] and found potential binding sites with multiple AS factors associated with GBM. Some of these factors promote tumorigenesis, such as SF2/ASF, hnRNPA1, hnRNPA2/B1, hnRNPF, hnRNPH1, YB1, NOVA1, PTB and so on. Some play tumor suppressor roles, such as SRp55 and MBNL. These factors may all be related to the production of HSD11B1L-181. After that, we will combine methods such as RNA immunoprecipitation, splicing minigene assays and site-directed mutagenesis to confirm the AS factors that have substantial influence.

In terms of GBM treatment, we may arrest HSD11B1L-181 expression by regulating the activity, quantity and localization of related splicing factors. The recent development is the treatment of specific decoy oligonucleotides that can target splicing factors and block their activity to correct the oncogenic isoform products of related genes [53]. Furthermore, at cis-regulatory elements, avoiding or enhancing the binding of specific splicing factors to target the pre-mRNA sequence also prevented the production of oncogenic isoforms. For example, use of antisense oligonucleotides to mediate AS switches causes exon skipping, premature stop codons and mRNA decay, resulting in diminished expression of oncogenic isoform [54]. It is worth mentioning that the interaction of a set of trans-activating splicing factors and their specific cis-regulatory elements is often involved in the AS of multiple genes. Therefore, cancer therapeutic strategies targeting AS mechanisms have the advantage of simultaneously correcting deficiencies in multiple gene targets. However, it may also interfere with the splicing of some genes unrelated to cancer. All of these are subject to a complete understanding of specific splicing factors and their recognition sequences being properly grasped. Therefore, the current therapeutic strategy to directly abolish the interaction between HSD11B1L-181 and parkin by establishing a small molecule drug (PMN) should be more feasible.

## 5. Conclusions

We found that *HSD11B1L* has a dominant splice variant HSD11B1L181 in GBM. Its overexpression can enhance the proliferation, migration and invasion potential of GBM cells. Knocking it down significantly blocked the cancerous properties of GBM cells. In the mechanism study, we confirmed that HSD11B1L-181 can interact with parkin protein, which may affect the activity of parkin. Furthermore, downregulation of parkin expression directly promotes the proliferation, migration and invasive properties of GBM. Finally, we found that PMN can abrogate the interaction between HSD11B1L-181 and parkin. We also confirmed the anti-GBM effect of PMN using cellular, heterotopic and orthotopic xenograft immunodeficient mouse models. This study not only provides a new direction for the study of GBM, but also proposes new opportunities for the therapeutic strategies of GBM.

## Data Availability

All data used and analyzed during the current study are available from the corresponding author upon reasonable request.

## Supplementary Materials

The following supporting information can be downloaded at: https://www.mdpi.com/article/10.3390/cells12060894/s1, Figure S1: Temozolomide and all 10 phytocompounds from our laboratory were assessed for their inhibitory effects on HSD11B1L-181 and parkin interactions using a yeast two-hybrid based growth assay. Log-phase cultures of diploid cells expressing BD-H2 and AD-P2 in the selective (-Leu-Trp-Ade- His) plates were spotted at different dilutions and incubated at 30 °C for 3 days.
