# Supplementary material for: Interaction of a Novel Alternatively Spliced Variant of HSD11B1L with Parkin Enhances the Carcinogenesis Potential of Glioblastoma: Peiminine Interferes with This Interaction
Source: Cells. 2023 Mar 14;12(6):894. doi: 10.3390/cells12060894 (PMC10047488; doi:10.3390/cells12060894)

Figure S1. Temozolomide and all 10 phytocompounds from our laboratory were assessed for their inhibitory effects on HSD11B1L-181 and parkin interactions using a yeast two-hybrid based growth assay. Log-phase cultures of diploid cells expressing BD-H2 and AD-P2 in the selective (-Leu-Trp-Ade- His) plates were spotted at different dilutions and incubated at 30 °C for 3 days.

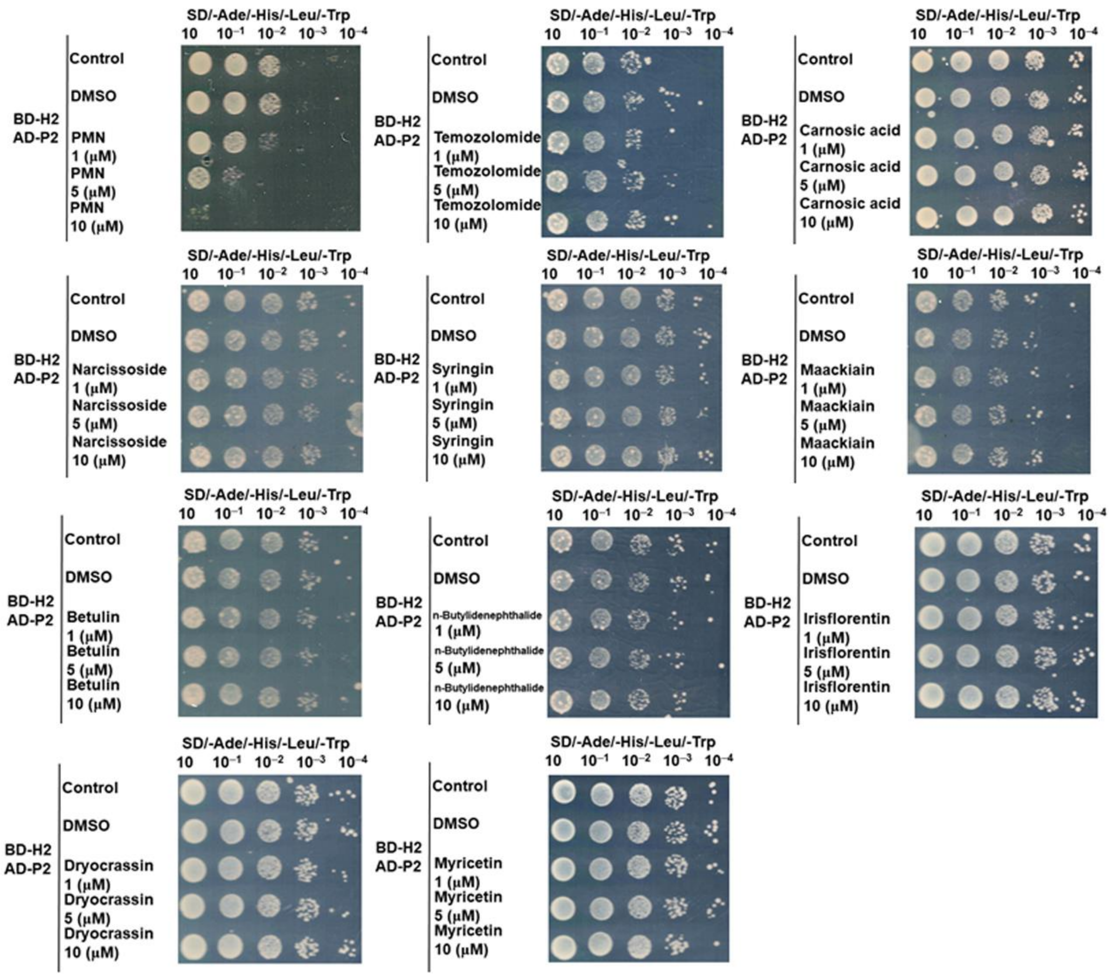

Supplement: Supplementary file 1 [file cells-12-00894-s001.zip › cells-2228781-supplementary.pdf]
